# Broadband Chiroptics with Twist‐stacked Hyperbolic Conducting Polymer Thin Films

**DOI:** 10.1002/adma.202417024

**Published:** 2025-03-12

**Authors:** Yulong Duan, Shangzhi Chen, Magnus P. Jonsson

**Affiliations:** ^1^ Laboratory of Organic Electronics Department of Science and Technology (ITN) Linköping University Norrköping SE‐601 74 Sweden

**Keywords:** chiral optics, conducting polymers, hyperbolic materials, subwavelength films

## Abstract

Chiral‐specific interaction of light with organic materials is important but typically arises from circular polarization‐dependent absorption of specific optical transitions, resulting in narrow effective wavelength ranges. This study presents a scalable and universal concept for broadband circular dichroism (CD) enabled by strained conducting polymer thin films that possess in‐plane hyperbolic optical behavior (i.e., optically metallic and dielectric properties along orthogonal directions). It is shown that off‐axis stacking of two or more such thin films provides broadband CD that varies with the hyperbolic bandwidth and stacking geometry. By contrast to traditional chiroptical materials, the CD can also be modulated by redox‐tuning of the hyperbolic polymer properties, opening for broadband dynamic chiroptical components.

## Graphical abstract

Anisotropic conducting polymer films can exhibit optical hyperbolic properties which here are found connected to large birefringence and linear dichroism. Off‐axis twist‐stacking of only two such subwavelength films can therefore enable large and broadband circular dichroism (CD), with CD bandwidth closely related to the hyperbolic bandwidth. The study demonstrates the general concept using twist‐stacked stretched PEDOT films.

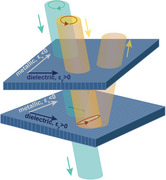

## 1 Introduction

The ability to produce, control, and detect chiral light is critically important across many fields, ranging from display technologies with enhanced efficiency and performance^[^ ^1^ ^]^ to lenses with negative refraction.^[^ ^2^ ^]^ Circularly polarized light also plays key roles in advancing areas such as quantum information processing,^[^ ^3^ ^]^ optical spintronics,^[^ ^4^ ^]^ and bioimaging.^[^ ^5^ ^]^ The latter pertains to the widespread presence of naturally occurring chiral molecules, whose molecular structures are dissymmetric and cannot be superimposed on their mirror images. Traditional approaches to controlling chiral light by chiral molecules have relied on the chiral absorption of their optical bands.^[^ ^6^ ^]^ However, this strategy is fundamentally limited to narrow bandwidths since it originates from specific absorption bands. Likewise, similar limitations apply also if chirality is induced by off‐axis stacking of non‐chiral thin films with linearly anisotropic absorption bands.^[^ ^7^ ^]^ We further note that the circular dichroism (CD) provided by natural chiral molecules is typically weak, with g‐factors on the order of 10^−3^ ((g_*A*_ = 2*(*A_R_* − *A_L_*)/(*A_R_* + *A_L_*) ), where *A_R_* and *A_L_* is the absorption of right‐handed and left‐handed polarized light, respectively).^[^ ^8^ ^]^ Stronger CD can be obtained using artificial microstructures or nanostructures acting as chiral optical antennas and metasurfaces,^[^ ^9^ ^]^ although typically requiring complex fabrication and with limited bandwidth when originating from resonance effects.

Beyond achieving large g‐factors and broadband response, novel chiroptical concepts preferably also offer added functionality, such as dynamic tunability or on‐chip control. Conducting polymers are promising in this regard as they provide both electrical conductivity and the ability to be tuned through their redox state. So far, conjugated polymer systems with chiroptical response have been achieved using chiral substituents, chiral self‐assembly or chiral templating.^[^ ^7^, ^10^ ^]^ However, the chiral response for those systems typically originated from the neutral optical absorption band of the polymer's low‐conductive state.^[^ ^11^ ^]^ Consequently, this leads to spectrally narrow CD similar to that of natural chiral molecules. Here, we here report a universal approach to generate strong and broadband CD based on the doped state of conducting polymers, at which they can provide an optically metallic spectral region and therefore a plasma frequency (ω_*p*_) at which the real part of the optical permittivity changes sign.^[^ ^12^ ^]^ Furthermore, this broadband CD can be switched off by de‐doping the polymer to its low conductivity state. Such tunable broadband CD holds significant potential for applications including white light displays, circularly polarized light detection, and broadband optical communication or data encryption.

At the core of the proposed concept is the ability to align the chains in a conducting polymer film to induce an in‐plane anisotropic ω_*p*_ ($\omega_{px}\neq \omega_{py}$) and therefore a spectral region where the film is optically metallic along one in‐plane optical axis while being dielectric along the other.^[^ ^13^ ^]^ Materials exhibiting such optical properties are known as hyperbolic optical materials, which have attracted significant research interest due to their potential applications in various fields.^[^ ^14^ ^]^ The reason for anisotropic ω_*p*_ for aligned conducting polymer films is attributed to the charge carriers having different effective masses along and perpendicular to the polymer chains.^[^ ^15^ ^]^ In turn, such anisotropic effective mass can be understood through the (inverse) relation between carrier mass and mobility, where the latter is more widely known to be anisotropic for aligned conducting polymer thin films.^[^ ^16^ ^]^ Here, we show that the in‐plane hyperbolic behavior of a conducting polymer thin film leads to strong linear birefringence (LB) and linear dichroism (LD) that overlap spectrally over a broad range. Analogous to other linearly anisotropic thin films,^[^ ^7a,c^ ^]^ chirality can then be induced by twist‐stacking two or more strained conducting polymer thin films. Owing to the hyperbolic permittivity of each film, this leads to broadband CD which can be adjusted via stacking geometry. Furthermore, the chiroptical response can be dynamically tuned by varying the polymer redox state. We demonstrate this concept using mechanically strained thin films of poly[3,4‐ethylenedioxythiophene:Sulfate] (PEDOT:Sulf) and discuss its applicability to other conducting polymers that can be aligned and exhibit spectral regions of negative permittivity.

## 2 Results and Discussion

**Figure 1:**
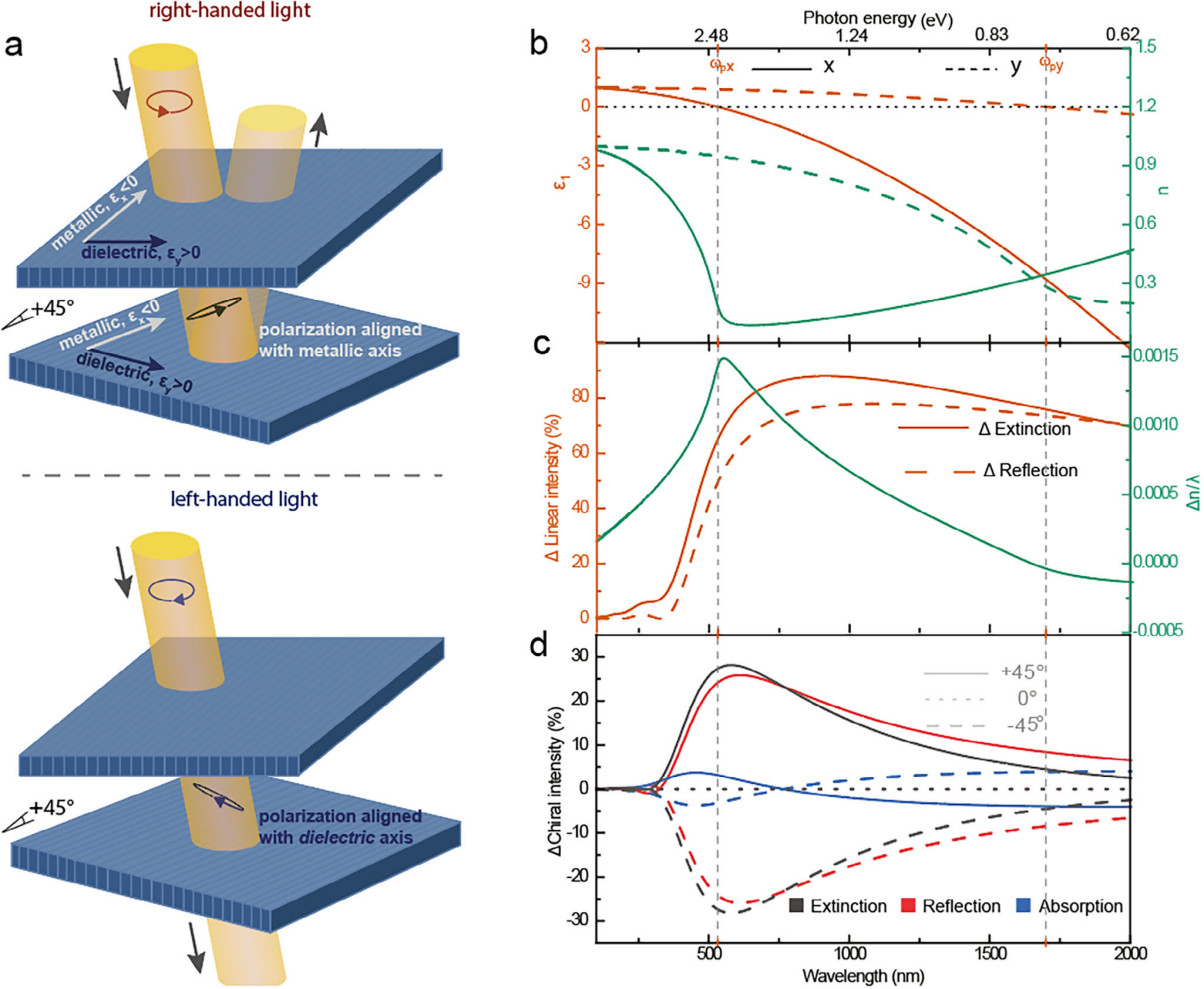
The concept of CD generation by twist stacking hyperbolic thin films. a) Illustration of the concept of generating CD from off‐axes twist‐stacking of two hyperbolic thin films. The large LB of the first hyperbolic film induces phase retardation and modification of the polarization state from chiral to elliptical. The angle of the elliptical major axis depends on the chirality of the incident light, which means that the strength of the polarization component that becomes aligned with the metallic direction of the second thin film will be different for left‐handed and right‐handed light. This leads to CD in both reflection/transmission (light propagation direction indicated by the blue arrows) by the second layer due to its large LD in the hyperbolic region. Note that the light is normal to the plane of the films in the simulations and measurements, while depicted at a non‐normal angle of incidence in the illustration to more easily distinguish incident and reflected beams. b) The real components of the in‐plane permittivity and refractive index of a hyperbolic material created using the Drude model with different values of m* along the two optical axes (anisotropy ratio for m* of 2.0). c) Normalized LB (expressed as Δ*n*/λ) for the same material as in (b) and LD (reflection and extinction) for a 200 nm thin film of the material. Both effects are enlarged near the hyperbolic wavelength region. d) Difference in extinction, reflection, and absorption between RCP light and LCP light for two 100 nm thick films twist‐stacked at ±45° angle or without twist (0°).

We theoretically illustrate the concept using in‐plane hyperbolic thin films with permittivity generated by the Drude model (**Figures** **1** and S1, Supporting Information) and then use the finite‐different time‐domain (FDTD) method to simulate the optical behavior for twist‐stacked films. To induce hyperbolic properties, we set the effective mass of the charge carriers (m*) to be anisotropic. As detailed in Note S1, m* affects the plasma frequency at which the material becomes metallic, which for anisotropic m* will occur at different spectral positions. This forms a hyperbolic spectral region between these positions. The hyperbolic bandwidth can then be conveniently varied via the strength of the anisotropy (Figures S2,S3 and Note S2, Supporting Information). Notably, hyperbolic properties cannot be induced by instead only assuming an anisotropic carrier mobility, because the mobility does not affect *ω*_p_. Likewise, the carrier density affects *ω*_p_ but cannot be anisotropic. Hence, the only means to induce hyperbolic properties for a Drude metal is via an anisotropic effective mass. In the hyperbolic range, the thin films have metallic properties along one in‐plane principal axis (negative real permittivity, ε_1_ < 0) while they possess dielectric properties along the other direction (positive real permittivity, ε_1_ > 0).^[^ ^14^ ^]^ Figure 1c shows that this feature induces an exceptionally large anisotropic linear reflection and corresponding large reflection‐based LD (Δ*R_L_* = *R_x_*  − *R_y_*), in a broad range centered within the hyperbolic region. Likewise, the in‐plane hyperbolic properties induce a large LB, as defined as the difference in real refractive index between the two principal axes (Δ*n*  = *n_y_*  − *n_x_*, where $n_{x,y}^{2}=\frac{1}{2}\;\sqrt{\left(\varepsilon_{1(x,y)}^{2}+\varepsilon_{2(x,y)}^{2}\right)}+\frac{1}{2}\;\varepsilon_{1(x,y)}$). The *n*_*x*,*y*_ dispersions show dips at respective plasma frequencies, at which they are determined only by the imaginary permittivity (*ε*_2_, Figure 1b). For the Drude‐based hyperbolic material, this causes a large broad LB centered near ω_*px*_ (Figure 1c).^[^ ^17^ ^]^ We here plotted the normalized LB (Δ*n*/λ) since it forms a wavelength‐independent evaluator for the radian phase retardation induced for a thin film (*R*  = *t*Δ*n*/λ , where *t* is the film thickness). Importantly, the LD and LB of the hyperbolic thin film show large spectral overlap. This is not an obvious result but due to the significant contribution from anisotropic reflection to the LD. As a comparison, a LD based purely on anisotropic absorption from an optical band transition would be related only to the imaginary refractive index (*k*) and the peak of Δ*k* would be positioned where Δ*n* is close to 0 (Figure S5 and Note S4, Supporting Information).

Thanks to the large and overlapping LB and LD, off‐axis stacking of two in‐plane hyperbolic films of subwavelength thickness can induce broad CD that covers and extends beyond the hyperbolic wavelength region. Figure 1d and S1 exemplify this using FDTD simulations for two films twist‐stacked at 45°, showing chiral extinction (Δ*E_C_* = *E_RCP_*  − *E_LCP_*, with RCP and LCP denoting incident light with right‐handed and left‐handed circular polarization, respectively) of several percent in the whole visible range and far into the near‐infrared range. The chiroptical response reverses if changing the twist direction from clockwise to anticlockwise, and it disappears at 0° twist. The ability to provide such a broad CD bandwidth originates from the hyperbolic properties of the films, as corroborated by a tight connection between the bandwidth of the CD and the bandwidth of the hyperbolic range (Figures S2,S3, Supporting Information). In short, the first film introduces phase retardation which induces polarization‐dependent reflection by the second film. Hence, the effect is based on strong chiral reflection (Δ*R_C_* = *R_RCP_*  − *R_LCP_*), while chiral absorption (Δ*A_C_* = *A_RCP_*  − *A_LCP_*) contributes only to a small extent in the lower wavelength regime (Figure 1d). This forms a distinguishing factor from chiral organic thin films with CD based on chiral absorption due to optical transitions.^[^ ^7^, ^11^ ^]^ For our concept, the contribution from Δ*A_C_* decreases (increases) if increasing (decreasing) *μ* (which controls the ratio between reflection and absorption, Note S3, Figure S4, Supporting Information). The important role of chiral reflection provides similar effects as that of chiral photonic crystals,^[^ ^18^ ^]^ but the presented concept provides a broadband response, only requires two layers, and is based on conducting (and redox‐tunable) materials.

**Figure 2:**
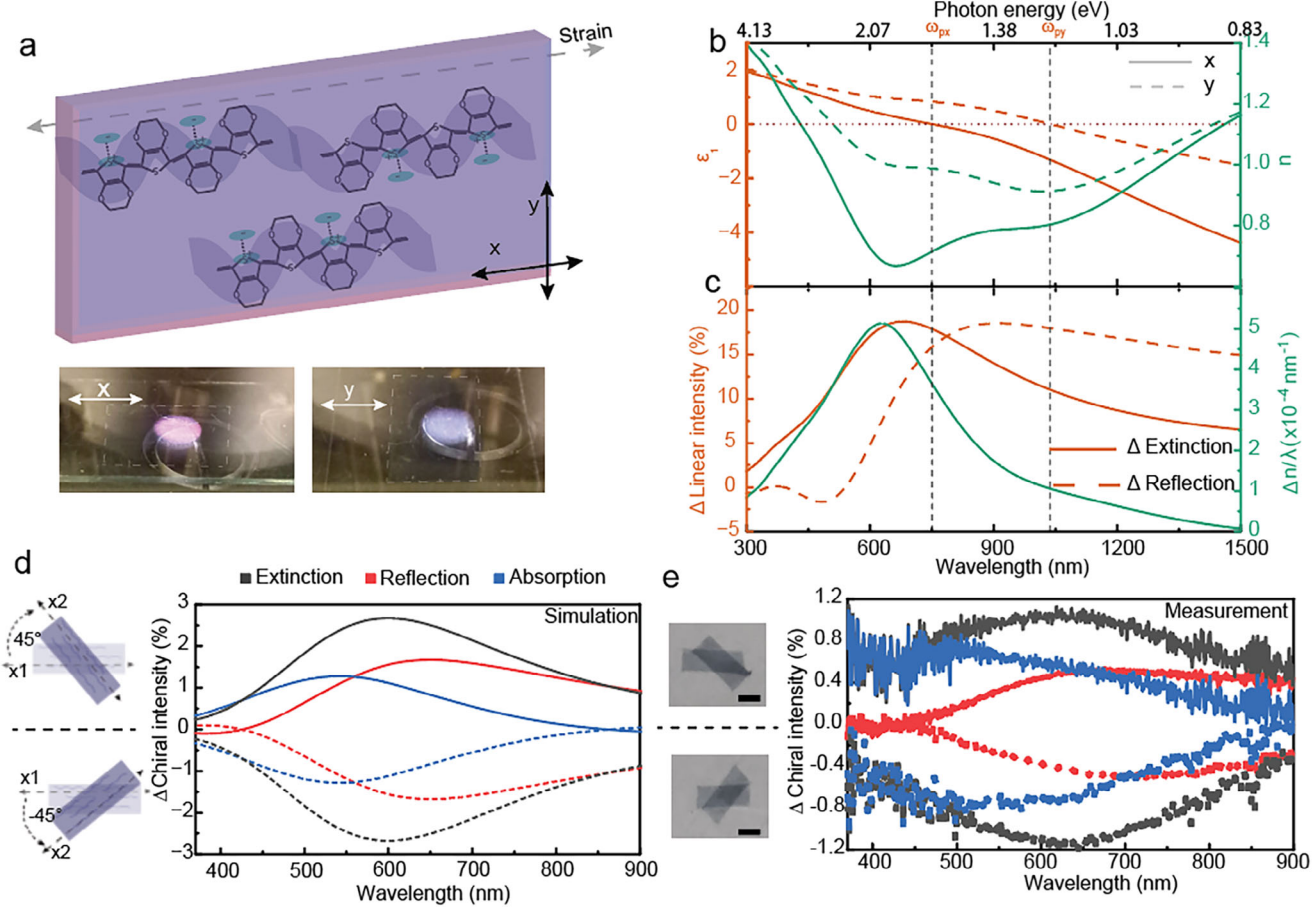
Experimental demonstration using strained conducting PEDOT films. a) Illustration of an anisotropic hyperbolic conducting polymer film. The PEDOT chains are partially aligned along the stretching direction (*x*). The bottom pictures are photos taken under normal light irradiation with polarization parallel (left) or perpendicular (right) to the strain direction. The white dashed boxes indicate the approximate sample areas. b) Real components of the in‐plane permittivity and refractive index of a 25% strained PEDOT: Sulf film. c) In‐plane specific linear birefringence (expressed as Δn/λ) and linear extinction and reflection dichroism dispersion. The dashed lines indicate the position of *ω*_px_ and *ω*_py._ d,e) Simulated (d) and measured (e) extinction (black lines), reflection (red lines) and absorption (blue lines) chiral intensity for two 25%‐strained PEDOT:Sulf films twist stacked at 45° (solid lines) and ‐45° (dash lines), as shown in the left diagram and photos, where x1 is the stain direction of the bottom layer. The scale bars are 1 cm.

We experimentally demonstrate the concept using conducting polymer films made anisotropic by uniaxial straining. This process aligns the polymer chains along the stretching direction, as previously used to obtain anisotropic conductivity owing to larger μ along the polymer chain direction compared to its normal direction.^[^ ^19^ ^]^ Less explored is that alignment can also lead to in‐plane anisotropic m*,^[^ ^13^, ^15^, ^16^ ^]^ which we here use to obtain anisotropic plasma frequency and in‐plane hyperbolic behavior. We chose poly(3,4‐ethylenedioxythiophene) (PEDOT) as the model polymer because it can be made sufficiently conducting to provide a transition from positive to negative ε_1_ in the visible or near‐infrared region.^[^ ^12^, ^13^, ^20^ ^]^ For simplicity, we refer to this transition as the plasma frequency although the match may not always be exact. **Figure** **2**b shows the measured permittivity for a vapor‐phase polymerized (VPP) PEDOT film doped by sulfuric acid (PEDOT: Sulf) and strained to 25% (see Methods for details). The results reveal a distinct 280 nm wide in‐plane hyperbolic permittivity region, owing to a considerably larger ω_*p*_ along the stretching direction (ω_*px*_ = 1.65 eV, at ≈750 nm) relative to the direction normal to the strain (ω_*py*_ = 1.20 eV, at around nm, Figure 2b). The hyperbolic permittivity results in anisotropic LD (Figure S8, Supporting Information) and cause anisotropic “metallic luster”^[^ ^21^ ^]^ that can be seen from photographs of the same film captured by irradiation with different linearly polarized light (Figure 2a). With the relationship of $\omega_{p(x,y)}=\sqrt{ne^{2}/\varepsilon_{0}m_{\left(x,y\right)}^{*}}$ from the Drude model, the hyperbolic dispersion of the material can be understood from the material having an in‐plane carrier mass anisotropy ratio of ≈1.9 ($m_{y}^{*}/m_{x}^{*}$). This means that the effective mass is about twice as large perpendicular to the strain direction compared to along the strain direction. We also note a modification from a pure Drude response due to (non‐mobile) polarons ≈700–1000 nm,^[^ ^22^ ^]^ which leads to a double‐dip shape for the *n* dispersions (Figure 2b). As a result, the normalized LB shows a broad response as predicted using the ideal Drude material, but with a peak position blue‐shifted into the visible by ≈120 nm from ω_*px*_ (Figure 2c). Simulations could reproduce this behavior by complementing the Drude response with a polaronic Lorentz term (even when the Lorentz term itself is isotropic, see detailed discussion in Note S5 and simulated results in Figures S6,S7, Supporting Information). The polaron‐induced blueshift can be understood as the modification of the peak wavelength of the birefringent value relative to that obtained only by the Drude model. Likewise, also the LD shows a broad response that is blueshifted compared with that of a pure Drude material (Figure 2b). As a result, the strained film provides significant LB and LD that overlap throughout the whole visible range, despite the hyperbolic range not covering this whole spectral region. The fact that the polaron‐contribution blueshifts the effective range is important for application in the visible range because it is challenging to push the hyperbolic range of conducting polymers that far.

Optical simulations using the permittivity of the strained PEDOT: Sulf predict that twist‐stacking of two subwavelength films (80 nm) can induce clear chiral extinction (Δ*E_C_*) across the visible range, with significant contributions from both Δ*R_C_* and Δ*A_C_* (Figure 2d). The more distinct contribution from absorption compared to the results for the hyperbolic Drude material in Figure 1 is due to lower carrier mobility (Figure S4, Supporting Information) and the existence of polarons. As expected, the chirality reversed if reversing the stacking direction (±45°). Experimentally, we realized such twist‐stacked systems via a layer‐by‐layer transfer process (see photos in Figure 2e) and determined their chiral reflection, absorption, and extinction using an integrating sphere approach (Scheme S1). The measured spectra reproduce the simulated results and confirm broadband chiroptical response (Figure 2e and Figures S9,S10, Supporting Information). The lower experimental CD signals can be attributed to light scattering due to surface roughness or other non‐idealities of the produced films compared to the ideal simulated systems. We note in this respect that the surface roughness is caused by crystallization domains formed during VPP rather than induced by stretching.^[^ ^13^ ^]^ The chiral strength can be evaluated using the dissymmetry g‐factor (g  = 2*(*I_R_* − *I_L_*)/(*I_R_* + *I_L_*) ), where the intensity (*I*) refers to either extinction or reflection.^[^ ^23^ ^]^

Simulations and experiments show that the reflection g‐factor can be as large as in the order of 10^−1^ for this system while the extinction g‐factor is in the order of 10^−2^ (Figures S11,S12, Supporting Information). In comparison, the absorption g‐factor of natural chiral molecules is in the order of 10^−3^.^[^ ^8^ ^]^ The g‐factor of the presented system is comparable to those of high‐performing systems based on conjugated polymers in their neutral state while offering larger bandwidth and only requiring sub‐100‐nm thickness (see Table S1 for a summary of some typical chiral conjugated polymer systems),^[^ ^7a^ ^]^ and with potential for significant improvement via enhanced polymer alignment as discussed below.

**Figure 3:**
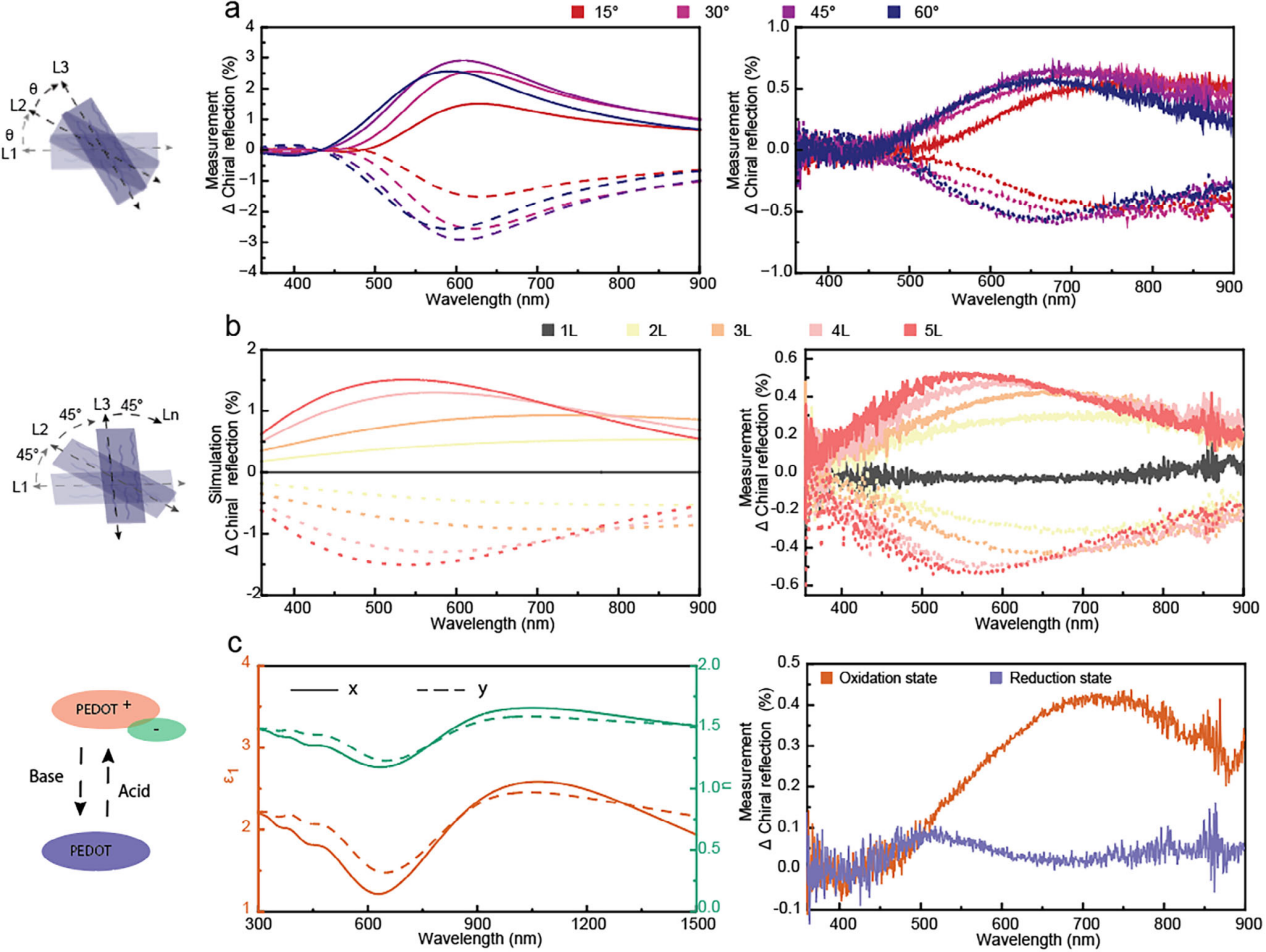
Tunable chiroptical response. a) Simulated (left) and experimental (right) ΔChiral reflection intensity between RCP and LCP on three‐layered samples with different twist angles (including clockwise (+θ) and anticlockwise (‐θ) stacking). The thickness of each layer was 80 nm. b) FDTD simulated (left) and experimental measured (right) reflection intensity difference between RCP and LCP with increasing layer numbers while keeping 45° twist between adjacent layers. The thickness of each layer is 40 nm. c) Real components of the in‐plane permittivity and refractive index of a 25% strained PEDOT: Sulf film after chemical reduction (left), and experimental measured chiral reflection intensity difference between RCP and LCP (right) for a two‐layer twist‐stacked system (45° twist and 80 nm layer thickness).

The chiral amplitude and effective spectral range of twist‐stacked hyperbolic films can be tuned via the twist angle or number of layers. These features are different from systems based on chiral molecules or chiral polymers, whose chirality is predetermined by their absorption spectra.^[^ ^7b^ ^]^ For three twist‐stacked hyperbolic PEDOT: Sulf films (each 80 nm thick), the position of maximum Δ*E_C_* and Δ*R_C_* blue‐shifted with increasing twist angle while the magnitude followed a non‐monotonic behavior (**Figures** **3**a,b and S13a,b, Supporting Information). The magnitude of the chiral reflectance was more than doubled by increasing the number of twist‐stacked layers from two to five, also accompanied with a blueshift (Figure 3c,d and Figure S13c,d, Supporting Information). These effects were all supported by optical simulations and can be explained by the modification of light polarization relative to the optical axes of the second layer when chiral polarized light passes through the first layer. This feature provides the ability to control the effective chiroptical wavelength range by changing stacking geometry, similar to for twisted 2D materials.^[^ ^24^ ^]^ Furthermore, the chiral response may be further modulated by the thicknesses of the LB and/or the LD layers, as the peak wavelength of the LB can maintain significant overlap with the LD for different film thicknesses (Figure S11a,b, Supporting Information). However, although increasing the thickness of the LB layer can increase phase retardation and eventually turn circular polarization into linear polarization, the g‐factor of the twist‐stacked system does not continuously increase with increasing thickness due to increasing absorption or reflection losses from the LB layer. Therefore, we here focused on thicknesses between 50–150 nm for the single layers (controlled by the polymerization time), which gave the largest LD and corresponding largest g‐factor for 45° twist stacked systems (Figure S11c,d, Supporting Information).

So far, our study focused on the chiral response when having the conducting polymer in its high‐conducting (hyperbolic) oxidized state. However, conducting polymers also offer dynamic tuning of their properties by altering the doping level via their redox state. This feature has been utilized in optical systems ranging from reflective displays^[^ ^25^ ^]^ and tunable plasmonic metasurfaces^[^ ^12^, ^20^ ^]^ to dynamic camouflage^[^ ^26^ ^]^ and radiative cooling systems.^[^ ^27^ ^]^ Here, we explore this feature for the twist‐stacked thin film concept by de‐doping the conducting polymer by exposure to the vapor of a highly branched poly(ethylenimine) (PEI).^[^ ^28^ ^]^ This procedure dramatically reduces the conductivity of the polymer such that the strained films are no longer hyperbolic but instead dielectric with positive permittivity along both *x* and *y* (Figure 3c). As anticipated, the removal of the hyperbolic properties largely reduced the LB (Figure S14, Supporting Information) and thus decreased the chiroptical response of the twist‐stacked films. By immersing the reduced twist‐stacked films in 1 M sulfuric acid, the CD properties could be restored to their original state due to the regeneration of the hyperbolic region during the re‐oxidation process (Figure S15a, Supporting Information). The CD of the twist‐stacked films in the oxidized state did not show any degradation even after 14 days, implying that the polymer alignment is stable (Figure S15b, Supporting Information). These results demonstrate the prospects for dynamically tunable devices, and also further highlights the critical role of the in‐plane hyperbolic permittivity for the chiroptical response.

A tight correlation between the hyperbolic properties and the chiral response of the twist‐stacked system was corroborated by modifying the position and/or bandwidth of the hyperbolic region using the Drude model and by varying m* along one or both in‐plane principal optical axes (Figures S2,S3 and Note S2, Supporting Information). The position of maximum Δ*n*/λ remained close to ω_*px*_ upon both blueshifts and redshifts of the hyperbolic region (Figure S2, Supporting Information). This provides an opportunity to control the spectral range of the CD without the need for varying sizes of nanostructures.^[^ ^18b^ ^]^ Importantly, increasing the hyperbolic bandwidth (by increasing the anisotropy of m*) extended the effective spectral region of the chiroptical response and also significantly increased its magnitude (Figure S3, Supporting Information). This is due to a combination of widened and enlarged LB as well as increased anisotropic reflection and absorption. Excitingly, conducting polymers indeed have the potential to provide such improvements by increasing the degree of polymer alignment. Density functional theory calculations suggest that the anisotropic ratio of m* can be as large as 36.9 for heavily doped crystalline PEDOT,^[^ ^15^ ^]^ which is close to 20 times higher than for the current system. Experimentally, the potential for large anisotropy is corroborated also by conductivity measurements, showing 1.5 ratio for the in‐plane conductivity of 25%‐strained PEDOT: Sulf^[^ ^13^ ^]^ while the ratio between in‐plane and out‐of‐plane conductivity can be more than 20.^[^ ^20c^ ^]^ The reason for the latter is a natural difference in chain orientation within and perpendicular to the plane for VPP PEDOT, leading to poor conductivity along the vertical direction relative to the in‐plane direction. This induces out‐of‐plane hyperbolic properties that are extremely broadband, starting from the visible and extending all the way into the far‐infrared region.^[^ ^12^ ^]^ To evaluate the potential chiroptical response from twist‐stacked systems of conducting polymers with a higher degree of alignment, we simulated the response for a material with permittivity along *y* set equal to the measured out‐of‐plane permittivity (*z‐*direction) (Figure S16, Supporting Information). The results for a two‐layer 45° twist‐stacked structure (100 nm thick films) reveal an extremely broad CD extending from 0.4 to 50 *µ*m. Therefore, it is reasonable to anticipate that improved in‐plane alignment of conducting polymer films will further enhance the CD effect and enlarge the bandwidth. Combined with the fact that the concept does not require nanostructuring, this result shows promise for upscaling and practical use in real applications.

In conclusion, we demonstrate a novel approach to generate dynamically switchable broadband chiroptical response based on the in‐plane hyperbolic properties of aligned PEDOT:Sulf thin films. The effective spectral range correlates with the hyperbolic region of the permittivity dispersion, making the concept universal and applicable to any hyperbolic materials that can be twist‐stacked. Likewise, we showed that enhancing the degree of in‐plane anisotropy can significantly enhance the bandwidth and strength of the hyperbolic response. It is worth noting in this respect that several other conducting polymers than PEDOT:Sulf have also shown negative permittivity regions, including poly(3‐hexylthiophene‐2,5‐diyl) (P3HT),^[^ ^29^ ^]^ polyaniline (PANI),^[^ ^30^ ^]^ poly(2,3‐dihydrothieno‐1,4‐dioxin)‐poly(styrenesulfonate) (PEDOT:PSS),^[^ ^20b^ ^]^ poly(2,5‐bis(3‐alkylthiophen‐2‐yl)thieno[3,2‐b]thiophene)  (PBTTT)^[^ ^31^ ^]^ and poly(benzodifurandione) (PBFDO).^[^ ^32^ ^]^ Due primarily to differences in charge density, the plasma frequency of these conducting polymers may be positioned at different wavelengths, which means that they may also provide different hyperbolic spectral regions after alignment. For example, the plasma frequency of lower charge density polymers like PEDOT:PSS is typically in the near‐infrared region, so the hyperbolic wavelength region can also be expected to be in the near‐infrared if such polymers are aligned. Future work may study their ability to form aligned systems and in particular to what extent such alignment will induce anisotropic carrier mass and hyperbolic behavior.

## 3 Materials and Methods

### 3.1 Optical Numerical Simulations

The numerical simulations were performed using the finite‐difference time‐domain FDTD) method using the commercial software Ansys Lumerical FDTD (https://www.ansys.com/products/photonics/fdtd). The mesh was 3 nm in all directions. The light propagated along the *z* direction, so periodic boundary conditions were used along the *x* and *y* directions while perfectly matched layer boundaries were used in the *z* direction. To generate a chiral light source, we used two plane wave light sources (period/bosh) at the same frequency region and same height. For LCP, the first light source was set at 0° polarization and 0° phase, and the second light source was set at 90° polarization and 90° phase. For RCP, the phase of the second light source was changed to ‐90° while other parameters were the same. Plane monitors were used, one above the light source to acquire reflection spectra (*R*) and one below the substrate to acquire transmission spectra (*T*). Extinction spectra (*E*) were obtained from 100%‐*R*‐*T*, and absorption spectra were calculated as *E*‐*R*. The chirality expressed as ΔIntensity was calculated from *I*_RCP_‐*I*_LCP_.

### 3.2 Fabrication of Twist Stacked Thin PEDOT:Sulf Films

SEBS substrates (H1052 from Asahi Kasei) were prepared by spin‐coating a SEBS solution (400 mg ml^−1^ in toluene) onto a clean glass substrate at a speed of 700 r.p.m. for 1 min, and then thermal annealing at 65 °C on a hot plate for 2 h to remove the solvent. Oxygen plasma treatment was used to modify the surface of as‐prepared SEBS to become hydrophilic. Oxidant solution was prepared by mixing 0.03 g Iron(III) trifluoromethanesulfonate (Fe(Otf)3) , 0.2 g PEG‐PPG‐PEG and 0.77 g ethanol. The oxidant solution was spin‐coated at 1000 rpm for 25–30s on the SEBS. Vapor phase polymerization (VPP) was conducted at a temperature of 60 °C inside a vacuum chamber. The thickness was controlled by the reaction time, from 40 min to 80 min. After polymerization, the as‐prepared samples were soaked in 99.7% ethanol for one hour and dried with nitrogen to remove excess oxidant. To improve the conductivity, the films were soaked in 1 m sulfuric acid for 40min. The resulting PEDOT: Sulf films on SEBS were then flipped to make the PEDOT side attach tightly on a clean glass substrate, and the two ends of the films were fixed to the glass substrate by tape. After that, by maintaining one end of the film fixed and unsticking the other tape, the film was uniaxially stretched to ≈25%. The amount of stretching was measured using a ruler marked on the substrate. The other end of the stretched film was then attached again to the glass substrate by the tape to maintain the strain. The strain was determined as the added length induced by stretching divided by the initial length (measured between the pieces of tape. The samples were then soaked in toluene to dissolve the SEBS. The tapes could then be removed, and the aligned PEDOT: Sulf films remained attached to the glass substrate. To get twist‐stacked films, we repeated the above procedure while controlling the angle of the strain direction between the layers.

### 3.3 Linear and Chiral Spectral Measurements

As depicted by Scheme S1, optical spectra were acquired using a setup based on a Xenon light source, an integrating sphere, and a spectrometer (Shamrock 303i, Newton detector, Andor Technology). A polarizer and quarter waveplate that covers the wavelength ranging from 350 to 900 nm were used to obtain chiral RCP or LCP light. Extinction (*E*) was measured by placing the sample at the light entrance of the integrating sphere (position 1 in Scheme S1) while reflection (*R*) was acquired by placing the sample at the light exit on the other side of the integrating sphere (position 2 in Scheme S1). To improve the signal‐to‐noise ratio for the chiral spectra, all extinction spectra and reflection spectra were averaged from 22 and 11 measurements of the same sample, respectively. Reflection spectra were calibrated with a 20% reflection standard reference, and air background was deducted in the final data. Absorption spectra (*A*) were obtained as *E*‐*R*. The chiral spectra (Δ*E*, Δ*R* or Δ*A*) were calculated by subtracting the LCP spectra from the RCP spectra of the same sample.

## Conflict of Interest

The authors declare no conflict of interest.

## Supporting information



Supporting Information

## Data Availability

The original data of this work are available via Zenodo at https://doi.org/10.5281/zenodo.13318776 or https://zenodo.org/records/13318776.
