# Supplementary material for: Broadband Chiroptics with Twist‐stacked Hyperbolic Conducting Polymer Thin Films
Source: Adv Mater. 2025 Mar 12;37(16):2417024. doi: 10.1002/adma.202417024 (PMC12016737; doi:10.1002/adma.202417024)
Supplement: Supplementary file 1 — Supporting Information [file ADMA-37-2417024-s001.pdf]

ADVANCED MATERIALS

Supporting Information

for *Adv. Mater.*, DOI 10.1002/adma.202417024

Broadband Chiroptics with Twist-stacked Hyperbolic Conducting Polymer Thin Films

*Yulong Duan, Shangzhi Chen and Magnus P. Jonsson**

Supplementary Materials for

Broadband Chiroptics with Twist-stacked Hyperbolic Conducting Polymer Thin Films

Yulong Duan, Shangzhi Chen and Magnus P. Jonsson*

Laboratory of Organic Electronics, Department of Science and Technology (ITN), Linköping University, SE-601 74 Sweden. E-mail: (magnus.jonsson@liu.se)

This PDF file includes:

Supplementary notes S1 to S5
Scheme S1
Figs. S1 to S16
Table S1

Supplementary Notes

Note S1 - Hyperbolic material generation using the Drude model

The Drude permittivity for metallic materials can be expressed as,

$$\varepsilon_{x,y,z}(\omega) = 1 - \frac{ne\mu_{x,y,z}}{\varepsilon_0(\omega^2\mu_{x,y,z}m^*_{x,y,z}/e + i\omega)}. \qquad (1)$$

Here, ε_0 is the vacuum permittivity, e is the electron charge, μ is the mobility of the charge carriers, τ is their mean scattering time, n is their density, and m^* is their effective mass. The subscripts indicate that μ and m^* are allowed to be different along the three principal optical axes, where we set x and y to be within the plane of the film and z orthogonal to the film. In turn, the position at which the real part of the permittivity (ε_1) changes sign from positive to negative is characterized by the plasma frequency

$$\omega_{p(x,y,z)} = \sqrt{ne^2/\varepsilon_0 m^*_{x,y,z}} \qquad (2)$$

, which will take different values along x and y if $m^*_x \neq m^*_y$. Hence, having anisotropic effective carrier mass can induce an in-plane hyperbolic region (between ω_{px} and ω_{py}). The in-plane anisotropic permittivity in Figure 1 and S1 generated by the Drude model have the following parameters: $m^*_x = 0.5m_0$ and $m^*_y = 5m_0$, where m_0 is the free electron mass. $\mu_x = 15\ \mathrm{cm^2 \cdot V^{-1} \cdot s^{-1}}$, $\mu_y = 2\ \mathrm{cm^2 \cdot V^{-1} \cdot s^{-1}}$ and $n = 2 \cdot 10^{21}\mathrm{cm^{-3}}$.

The dispersion of the imaginary part of refractive index k was obtained from the permittivity using:

$$k^2 = \frac{1}{2}\left(\sqrt{(\varepsilon_1^2 + \varepsilon_2^2)} - \varepsilon_1\right). \qquad (3)$$

Note S2 - Influence of hyperbolic bandwidth and spectral position

To evaluate the relationship between the CD and the hyperbolic wavelength region, we shifted or extended the hyperbolic wavelength region by using the Drude model and altering m^*_x and/or m^*_y while keeping other parameters fixed ($\mu_x = 15\ \mathrm{cm^2 \cdot V^{-1} \cdot s^{-1}}$, $\mu_y = 10\ \mathrm{cm^2 \cdot V^{-1} \cdot s^{-1}}$ and $n = 2 \cdot 10^{21}\mathrm{cm^{-3}}$).

As shown in Figure S2, the hyperbolic region gradually red shifts if increasing m^* along both in-plane directions. The materials had the following m^*: No.1: $m^*_x = 0.4\,m_0$ and $m^*_y = 0.8\,m_0$; No.2: $m^*_x = 0.5\,m_0$ and $m^*_y = 1.0\,m_0$; No.3: $m^*_x = 0.6\,m_0$ and $m^*_y = 1.2\,m_0$; No.4: $m^*_x = 0.7\,m_0$ and $m^*_y = 1.4\,m_0$; No.5: $m^*_x = 0.8\,m_0$ and $m^*_y = 1.6\,m_0$.
As shown in Figure S3, the hyperbolic region gradually extended its bandwidth if decreasing m^*_x while increasing m^*_y (increasing the anisotropy ratio). The materials had the following m^*: No.1: $m^*_x = 0.7\,m_0$ and $m^*_y = 0.8\,m_0$; No.2: $m^*_x = 0.6\,m_0$ and $m^*_y = 0.9\,m_0$; No.3: $m^*_x = 0.5\,m_0$ and $m^*_y = 1.0\,m_0$; No.4: $m^*_x = 0.4\,m_0$ and $m^*_y = 1.1\,m_0$; No.5: $m^*_x = 0.3\,m_0$ and $m^*_y = 1.2\,m_0$.

Note S3 - Influence of charge carrier mobility

We altered the value of μ along both in-plane directions, keeping m^* and n fixed, to explore how the mobility of charge carriers influences the CD of twist films (results presented in Figure S4). We used the following parameters: $m_x^* = 0.5\, m_0, m_y^* = 1.0\, m_0$, and $n = 2 \cdot 10^{21}\text{cm}^{-3}$. The value of μ was gradually increased in both directions with the following values for the different materials: No.2: $\mu_x = 3.75\ \text{cm}^2 \cdot \text{V}^{-1} \cdot \text{s}^{-1}$ and $\mu_y = 2.5\ \text{cm}^2 \cdot \text{V}^{-1} \cdot \text{s}^{-1}$; No.2: $\mu_x = 12\ \text{cm}^2 \cdot \text{V}^{-1} \cdot \text{s}^{-1}$ and $\mu_y = 8\ \text{cm}^2 \cdot \text{V}^{-1} \cdot \text{s}^{-1}$; No.3: $\mu_x = 60\ \text{cm}^2 \cdot \text{V}^{-1} \cdot \text{s}^{-1}$ and $\mu_y = 40\ \text{cm}^2 \cdot \text{V}^{-1} \cdot \text{s}^{-1}$.

Note S4 - Band transition, the Lorentz model

A band transition can be expressed by the Lorentz model as:

$$\varepsilon_{x,y,z}(\omega) = \varepsilon_\infty - \frac{A_{x,y,z}}{\omega^2 - \omega_{x,y,z}^2 + i\omega\gamma_{x,y,z}} \qquad (4)$$

where A_L, ω_L, and γ_L are amplitude, resonance frequency, and broadening for the Lorentz oscillator, respectively. For the results presented in Figure S5, the resonance frequency was set to 2.07 eV (599 nm) with a broadening of 0.1. The amplitudes along the x and y direction were 0.2 and 0.4, respectively. ε_∞ is the high-frequency permittivity offset, which was set to 1 to maintain consistency with the Drude model.

Note S5 - Effects of band transition in a hyperbolic material, the Drude-Lorentz model.

For a hyperbolic material simulated with the Drude model, $\Delta n/\lambda$ relates to the width of the hyperbolic range and is maximized at the plasma frequency point along the metallic direction of the hyperbolic range (donated as ω_{px}). This is the reason that the effective CD wavelength in twisted films closely relates to the hyperbolic range. To examine whether band transitions influence the correlation between CD wavelength and the hyperbolic range, we modified the Drude component by adding a Lorentz oscillator, which forms the Drude-Lorentz model as:

$$\varepsilon_{x,,y,z}(\omega) = 1 - \frac{ne\mu_{x,y,z}}{\varepsilon_0(\omega^2\mu_{x,y,z}m_{x,y,z}^*/e + i\omega)} - \frac{A_{x,y,z}}{\omega^2 - \omega_{x,y,z}^2 + i\omega\gamma_{x,y,z}} \qquad (5)$$

We used the same parameters for the Drude component as those in Note S1 and Figure 1, for which ω_{px} and ω_{py} were located at 532 and 753 nm, and the peak of $\Delta n/\lambda$ was located at 545 nm near the ω_{px}.

If the frequency of the Lorentz oscillator is near the hyperbolic region, it can modify the plasma frequency positions relative to the Drude model. In addition, the presence of the Lorentz oscillator can also move the peak position of $\Delta n/\lambda$ out of the hyperbolic range or split the peak into two. This behavior is exemplified in Figure S6, in which a Lorentz oscillator (2.2 eV, 564 nm) with frequency inside the hyperbolic range was added to modify the Drude permittivity. The situation resembles that of a hyperbolic conducting polymer with the presence of a polaron transition. The amplitude of the Lorentz oscillator was varied from 0 to 0.4 for Materials No.1 to No.5, with a step size of 0.1 along both *x*- and *y*-directions. The results show that even an isotropic band transition near the hyperbolic range can modify the LB spectra, which can explain the blue shift of the measured LB peak relative to ω_{px} for the polymer material.

We also tested the possible effects of band transitions at longer wavelengths far from the hyperbolic region, but they did not significantly influence the position of ω_p and refractive index nearby. This means that such band transitions would exhibit negligible effects on the LB and LD near the hyperbolic range for a hyperbolic material. Figure S7 exemplifies this for the addition of a Lorentz oscillator at 8856 nm (0.14 ev), which is far away from both ω_{px} and ω_{py}. Even for large changes in amplitude (A_L, varied from 10 to 40), which induced abrupt permittivity changes near the Lorentz oscillator frequency, the oscillator showed negligible effects on the permittivity and refractive index near the hyperbolic region. Thus, such a band transition would not affect the result as that of the Drude model.

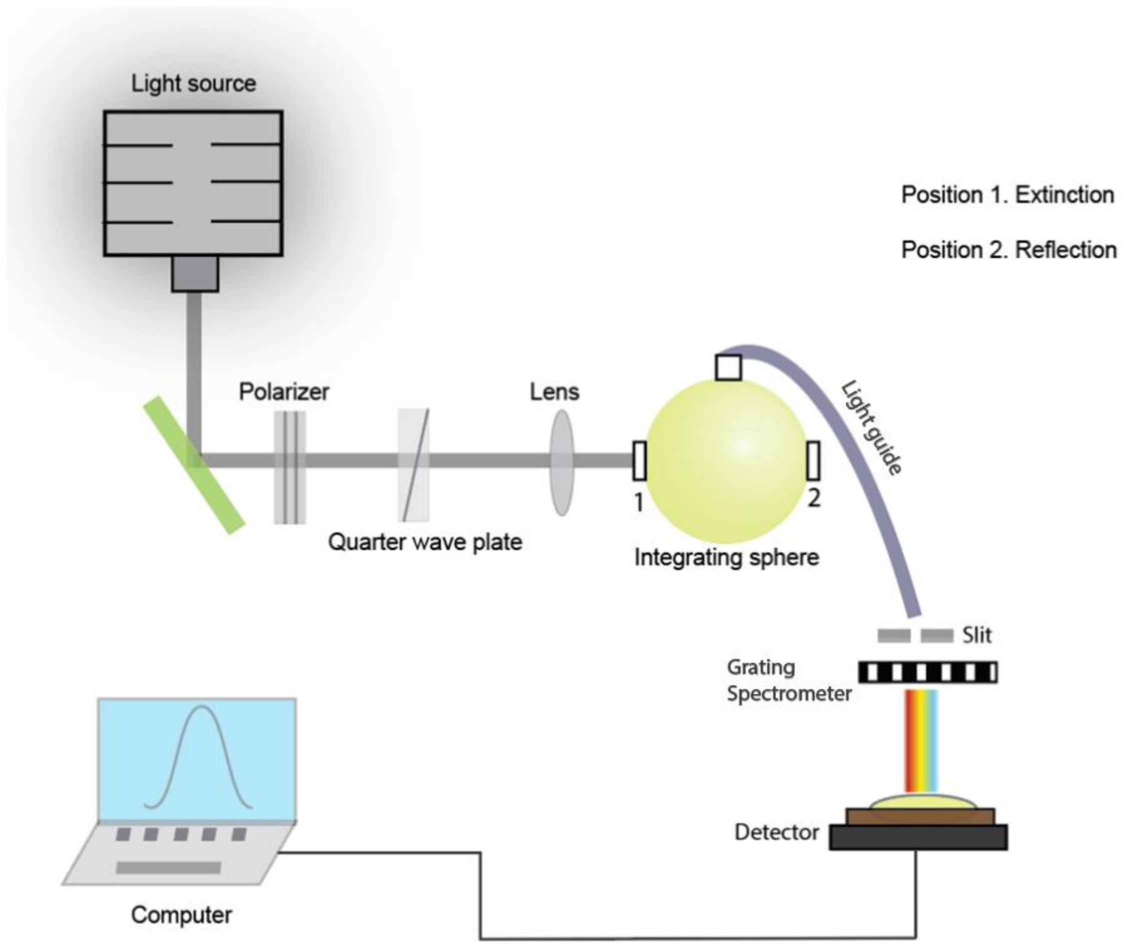

Scheme S1. Schematic illustration of experimental setup for acquiring chiral spectra.

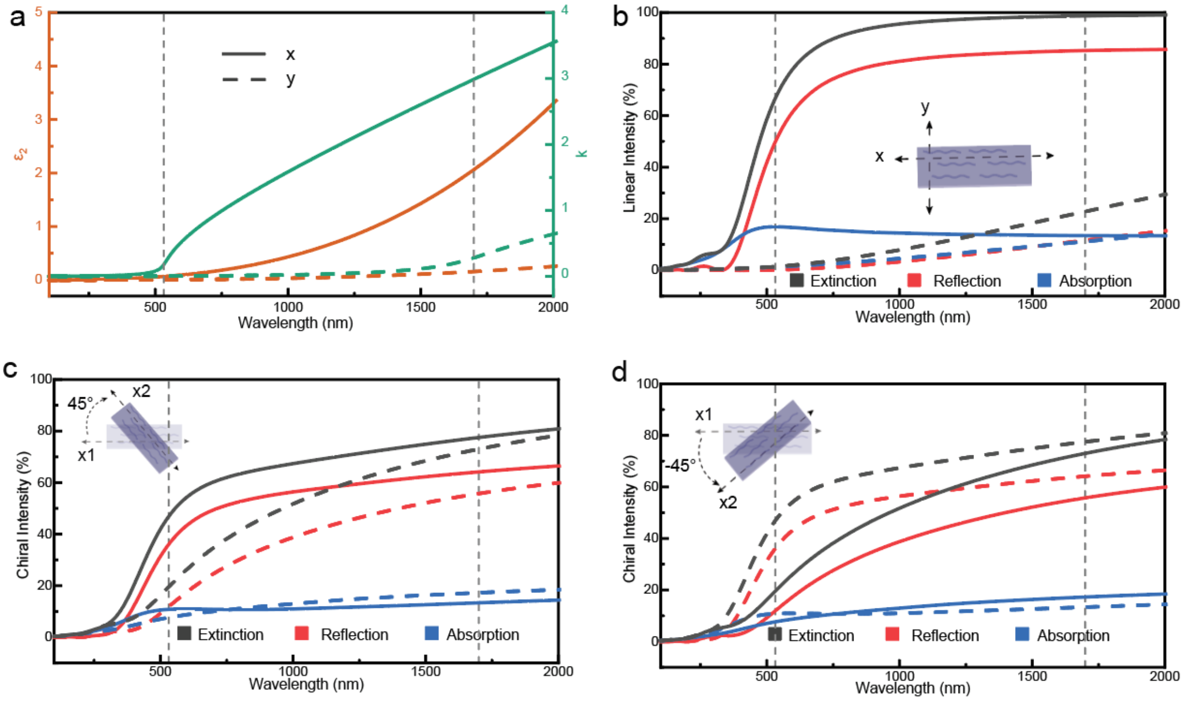

Figure S1. Simulated chirality in twist-stacked hyperbolic films. a ε_2 and *k* of a hyperbolic material generated from the Drude model with different values of *m** along the two in-plane optical axes. **b** Simulated linear polarized spectra of a single hyperbolic film (200 nm thickness). **c, d** Simulated chiral polarized spectra of two hyperbolic films (each 100 nm) twist-stacked at 45° (c) and -45° (d). The dashed lines in each panel indicate the two plasma frequency positions. The simulated parameters for Drude Model can be found in Note S1.

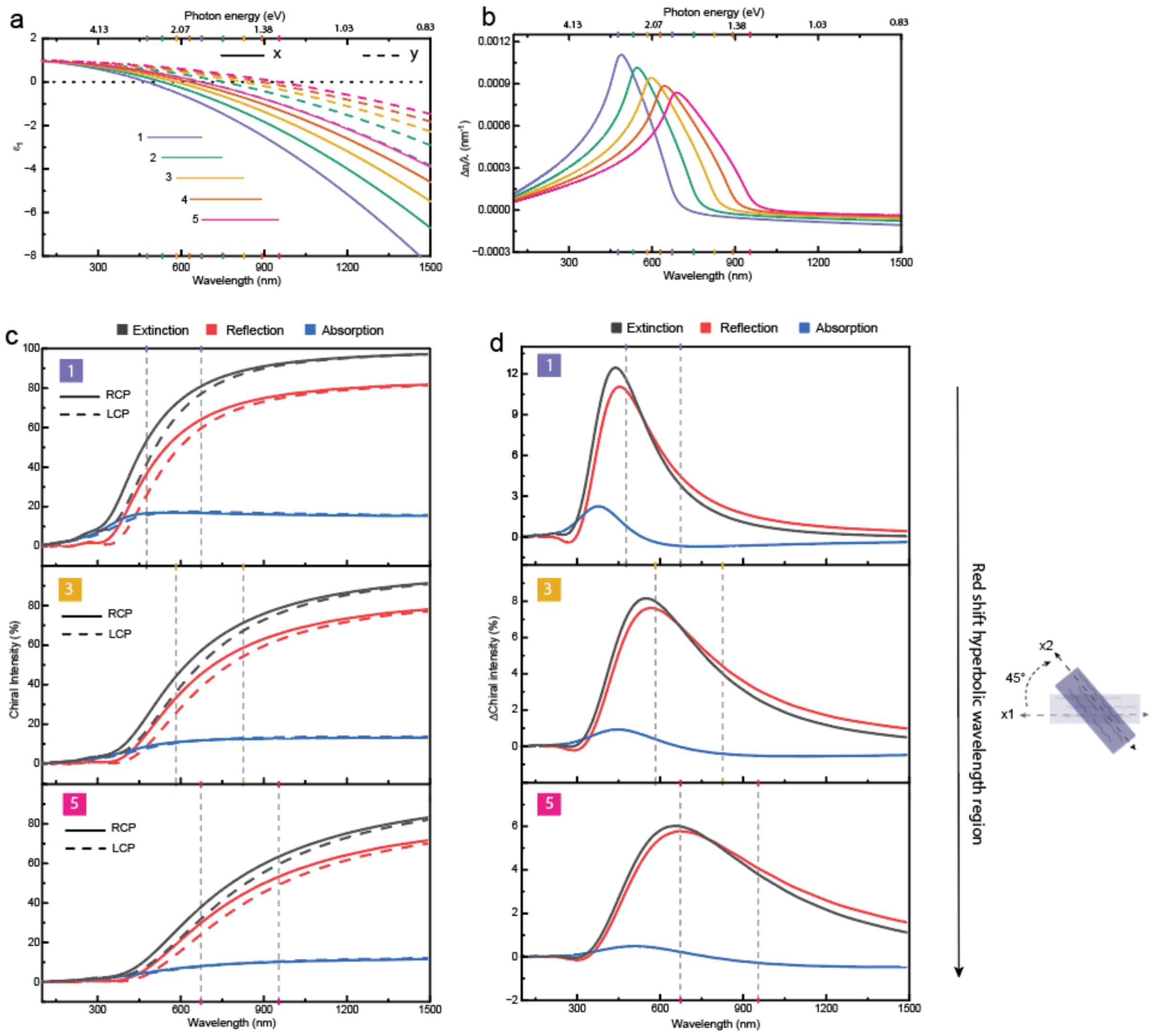

Figure S2. Simulated chirality of twist-stacked hyperbolic films for hyperbolic regions at different positions. a Red-shifting of the hyperbolic region by synchronous increase of m^* for materials No.1 to No.5 as described in Note S2 together with other parameters used. **b** The LB values ($\Delta n/\lambda$), which also show corresponding peak shift with the shifting of hyperbolic region. **c** Chiral spectra (left) and CD spectra (right) of two twisted films simulated with the permittivity of materials No.1, No.3 and No.5 as indicated in the panels. The two films, each thickness of 100 nm, were twisted at 45° as shown to the right, where x1 is the bottom layer. The results show that the effective CD wavelengths red-shift along with the red-shifting of the hyperbolic region. The dashed vertical lines in each panel indicate the two plasma frequency positions.

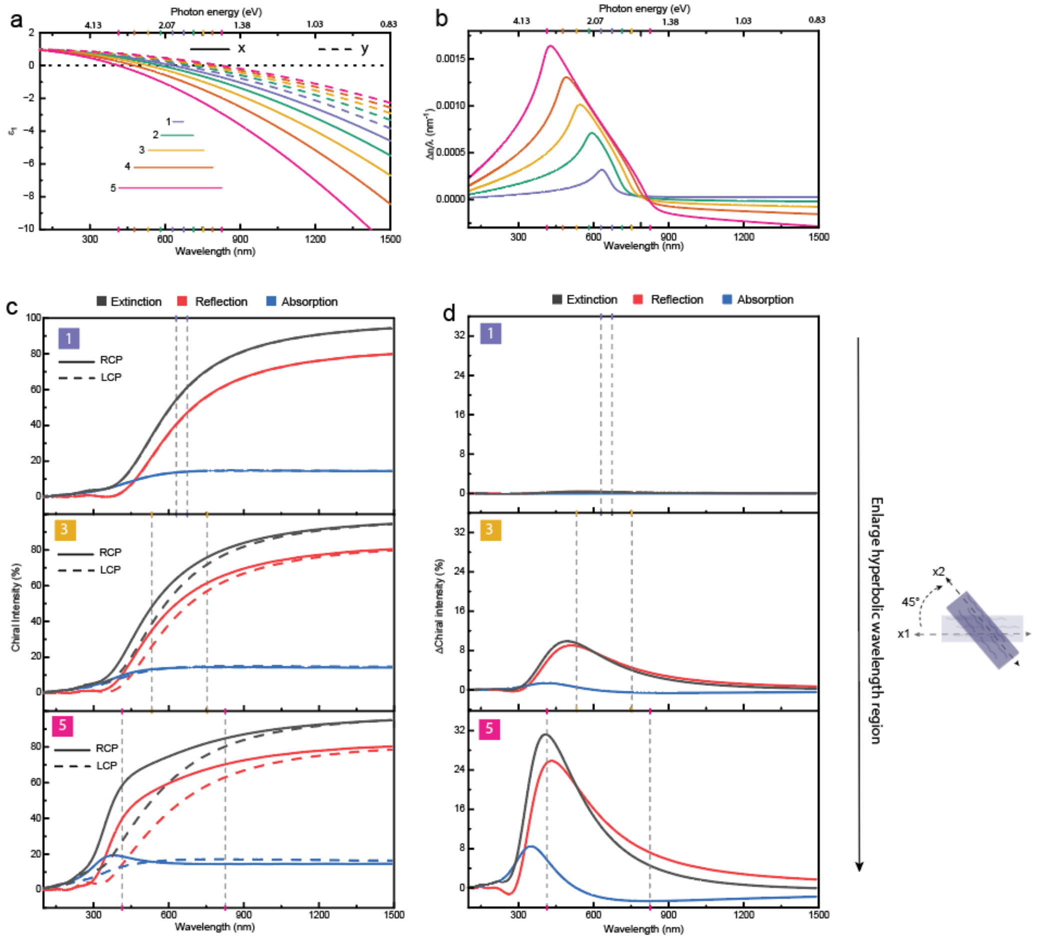

Figure S3. Chirality for twist-stacked hyperbolic films upon increasing the hyperbolic bandwidth. a Extending the hyperbolic region by decreasing the value of m^* along x while increasing it along y for materials No.1 to No.5 as described in Note S2 together with other parameters used. **b** The change the LB ($\Delta n/\lambda$), which shows one order of magnitude increase when extending the hyperbolic region. **c** Chiral spectra (left) and CD spectra (right) of two twisted films simulated with the permittivity of 1, 3 and 5 as indicated in the panels. The two films, each with thickness of 100 nm, were twisted at 45° as shown in the right, where x1 is the bottom layer. The results show that both the effective CD magnitude and wavelength range increase when extending the hyperbolic region. The vertical dashed lines in each panel indicate the two plasma frequency positions.

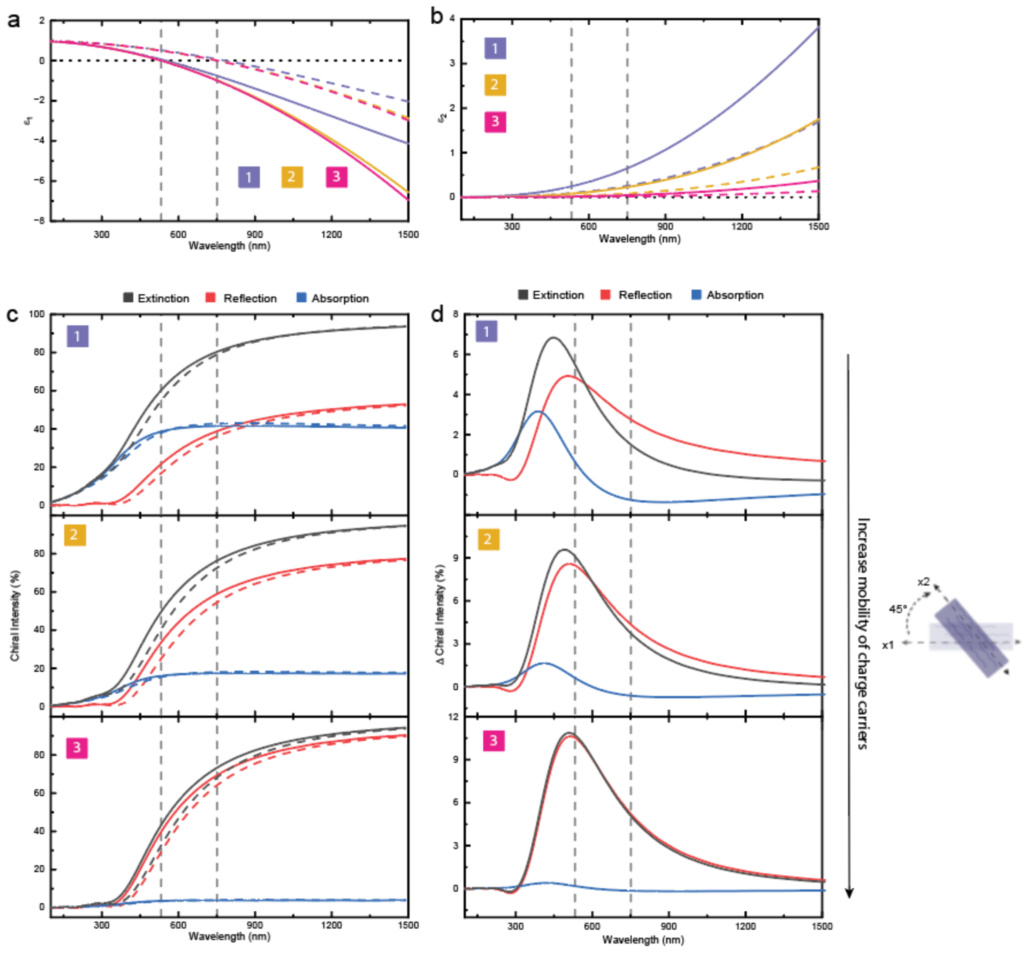

Figure S4. Simulated chirality for twist-stacked hyperbolic films by changing the charge carrier mobility. a, b Change in the real part (a) and imaginary part (b) of material permittivity with increasing the value of μ from 1 to 3. **c** and **d** Chiral spectra (c) and CD spectra (d) of two hyperbolic films (each 100nm) twist-stacked at 45°. The parameters for the Drude model can be found in Note S3. The results show that although the charge carrier mobility cannot directly affect the plasma frequency, increasing the charge carrier mobility can decrease chiral absorption while increasing chiral reflection. The vertical dashed lines in each panel indicate the two plasma frequency positions.

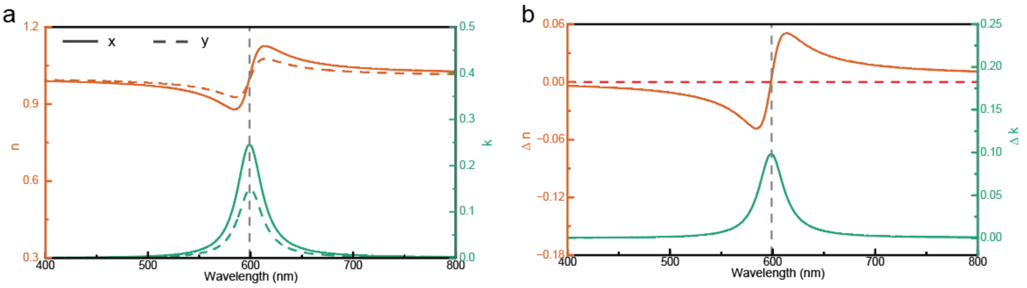

Figure S5. (a) Calculated refractive index and (b) corresponding refractive index differences of an anisotropic Lorentz oscillator with different amplitudes along the *x*-direction (0.2) and *y*-direction (0.4). The vertical grey dashed line indicates the wavelength location of the Lorentz oscillator at 1.07eV (599 nm). The horizontal orange dashed line in (b) indicates where Δn is 0. At the peak wavelength of Δk, Δn is approximately 0.

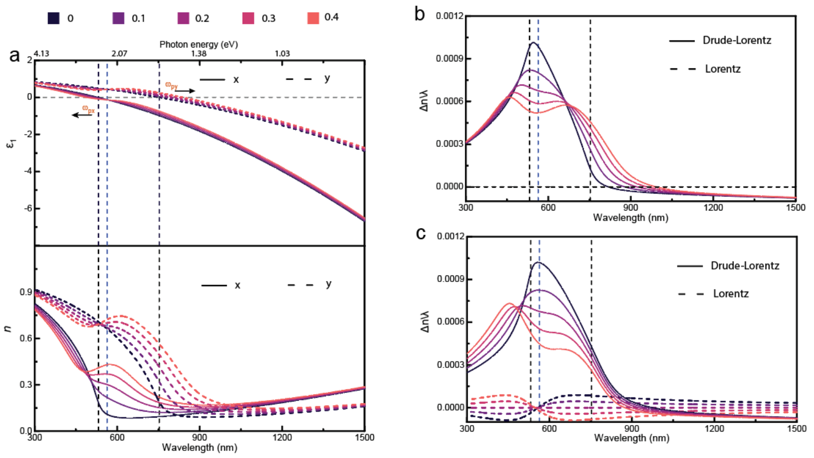

Figure S6. Drude-Lorentz model. a The real part of permittivity (top) and refractive index (bottom) near the hyperbolic region simulated with the Drude-Lorentz model by adding a Lorentz oscillator at 2.2 eV and varying its amplitude from 0.1 to 0.4. **b** Normalized LB ($\Delta n/\lambda$) for synchronous increasing amplitude of the Lorentz oscillator from 0 to 0.4 along both *x* and *y*. **c** Normalized LB ($\Delta n/\lambda$) by varying the amplitude of the Lorentz oscillator from 0 to 0.4 along *x* while keeping it fix at 0.2 along *y*. The solid lines in (b) and (c) correspond to the Drude-Lorentz model while the dashed lines correspond to only the Lorentz part. The vertical blue dashed lines show the wavelength location of the Lorentz oscillator. The vertical black dashed lines indicate the wavelength location of the plasma frequencies from the Drude part. The results show that a band gap transition near the hyperbolic region can modify the peak wavelength of the normalized LB and blue-shift it out from the hyperbolic region.

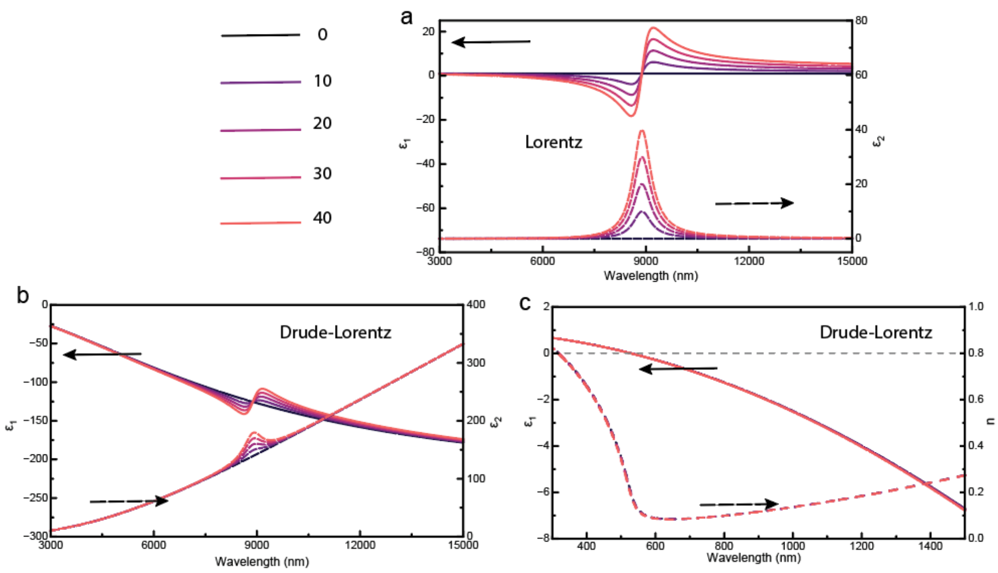

Figure S7. Permittivity generated from the Drude-Lorentz model with Lorentz oscillator at lower frequency far from the plasma frequency. a Permittivity generated by the Lorentz model for an oscillator located at 0.14 eV and with increasing amplitude from 0 to 40. **b** Permittivity near the Lorentz oscillator frequency generated from the Drude-Lorentz model (same Lorentz oscillators as in (a). **c** Real part of the permittivity and real refractive index near the plasma frequency generated from the Drude-Lorentz model (same Lorentz oscillators as in (a)). The results show that an oscillator at low frequency far from the plasma frequency has negligible effects on the permittivity and refractive index near the plasma frequency, also when the strength of the oscillator is large.

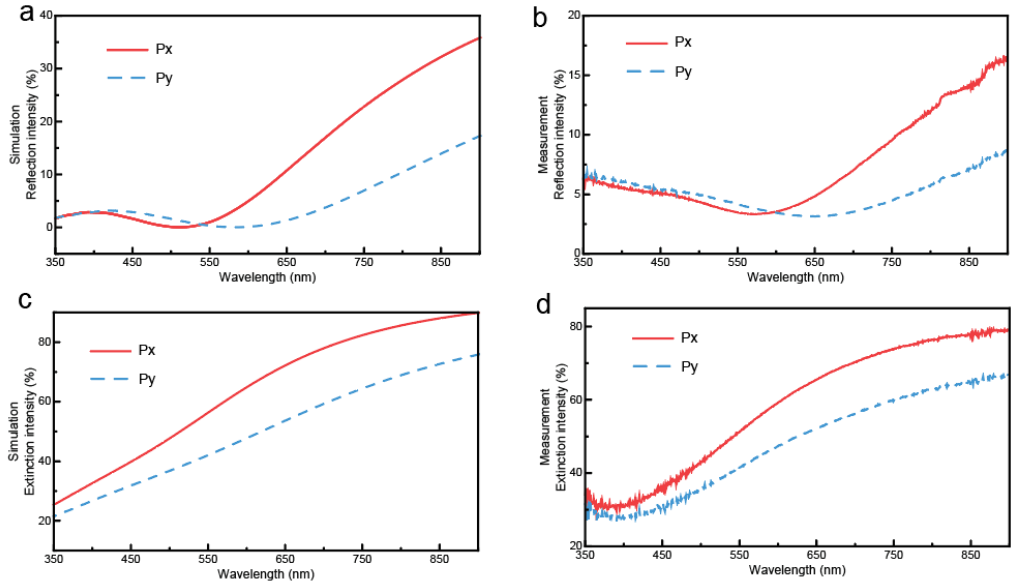

Figure S8. Simulated and measured linear polarized spectra of a 25% strained film on glass. a, b Simulated (a) and measured (b) reflection spectra. **c, d** Simulated (c) and measured (d) extinction spectra. The film thickness was 160 nm and the *x*-direction is the stretching direction. Note that although the dip in the reflection spectra is commonly used to assign the ω_p point, simulations using the Drude model reveal that in subwavelength thick films, this dip experiences a blue shift relative to the ω_p (Figure S1b). Therefore, the ω_{px} at 750 nm causes light reflection that extends into the visible spectral region, resulting in a 160 nm film exhibiting a red "metallic sheen" when irradiated with light parallel to the strained direction (photos in the bottom part of Figure 3a). On the other hand, the ω_{py} at 1030 nm causes lower reflection in the visible region, leading to the disappearance of the 'metallic sheen' when the sample is illuminated with light polarized normal to the strain direction.

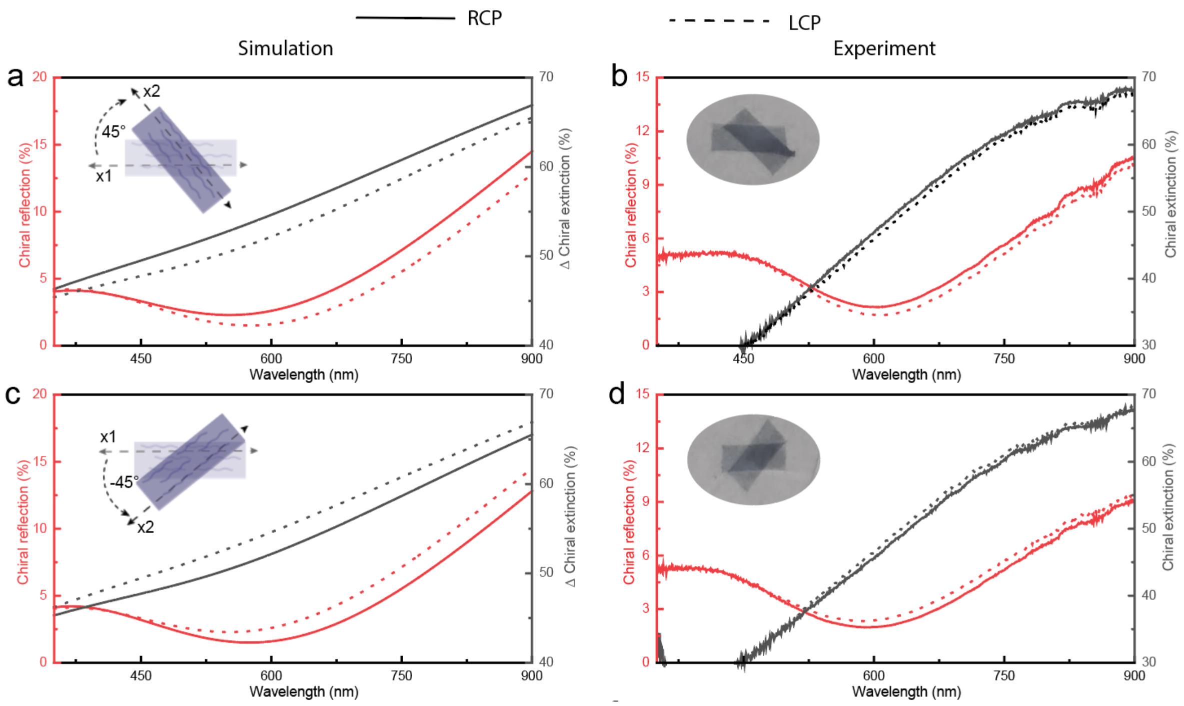

Figure S9. Chiral optical spectra of two 25% strained films (each 80nm) twist-stacked at 45° and -45°. a, b Simulated (a) and measured (b) chiral reflection and extinction spectra of two 45° twisted films. **c, d** FDTD simulated (c) and measured (d) chiral reflection and extinction spectra of two -45° twisted films. The inserts in (b) and (d) are photos of the stacked films with corresponding twist-stack directions as illustrated in (a) and (c), respectively.

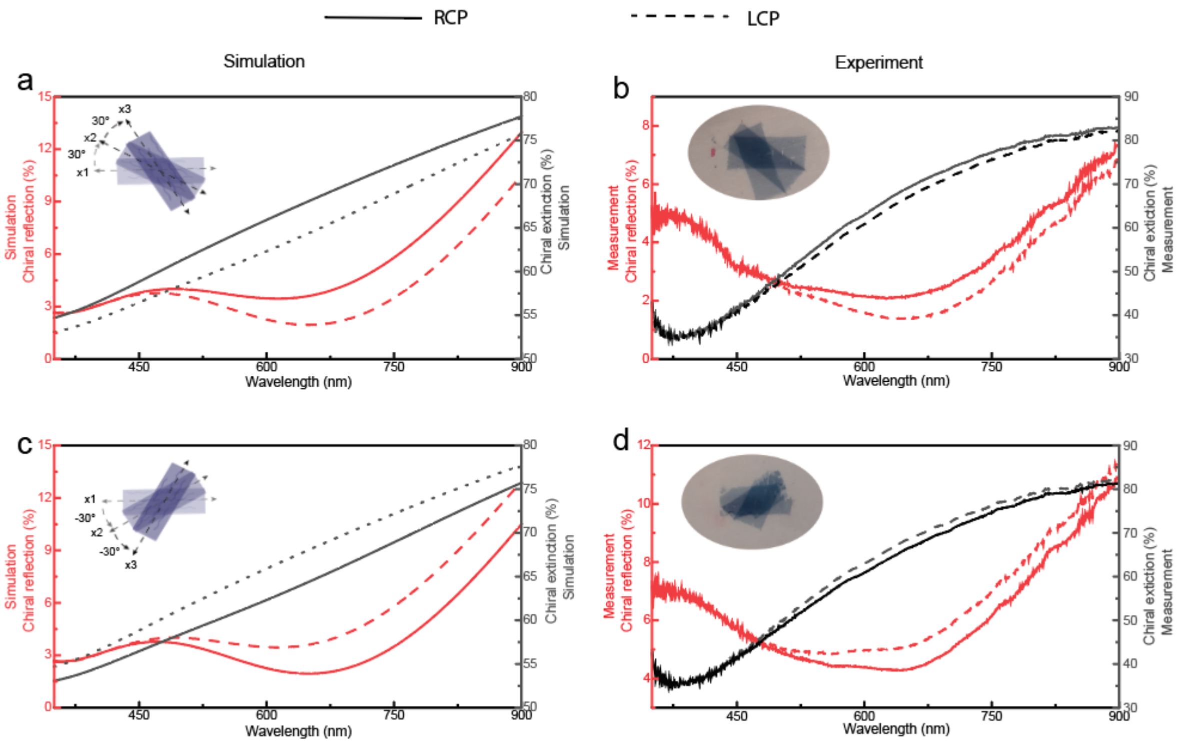

Figure S10. Chiral optical spectra of three 25% strained films (each 80nm) twist-stacked at 30° and -30°. a, b Simulated **(**a) and measured (b) chiral reflection and extinction spectra of three 30° twisted films. **c, d** FDTD simulated (c) and measured (d) chiral reflection and extinction spectra of three -30° twisted films. The inserts in (b) and (d) are photos of the stacked films with corresponding twist-stack directions as illustrated in (a) and (c), respectively.

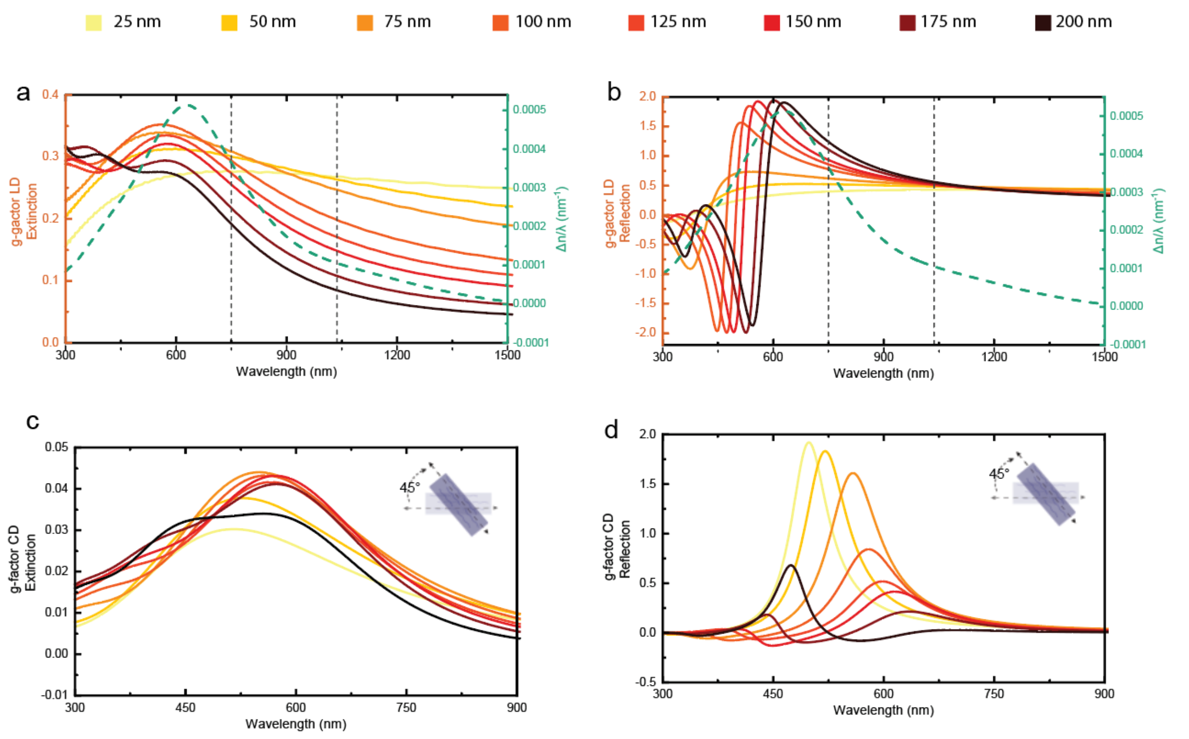

Figure S11. Film thickness optimization in a 45° two-layer twisted system by simulation. a, b LD extinction (a) and reflection spectra (b) with different film thickness. Although the LD spectra show good overlap with the LB in different film thickness, a film with 50-150 nm shows a lager LD value in extinction. The g-factor of LD value was calculated by $\frac{2(I_x - I_y)}{I_x + I_y}$, where I_x is the extinction or reflection intensity with linear polarization along the strain direction, while I_y is the corresponding spectra intensity normal to the strain direction. **c, d** CD g-factor of the extinction (c) and reflection (d) in a 45° twisted two-layers system, by changing LB layer thickness while fixing the LD layer as 100 nm.

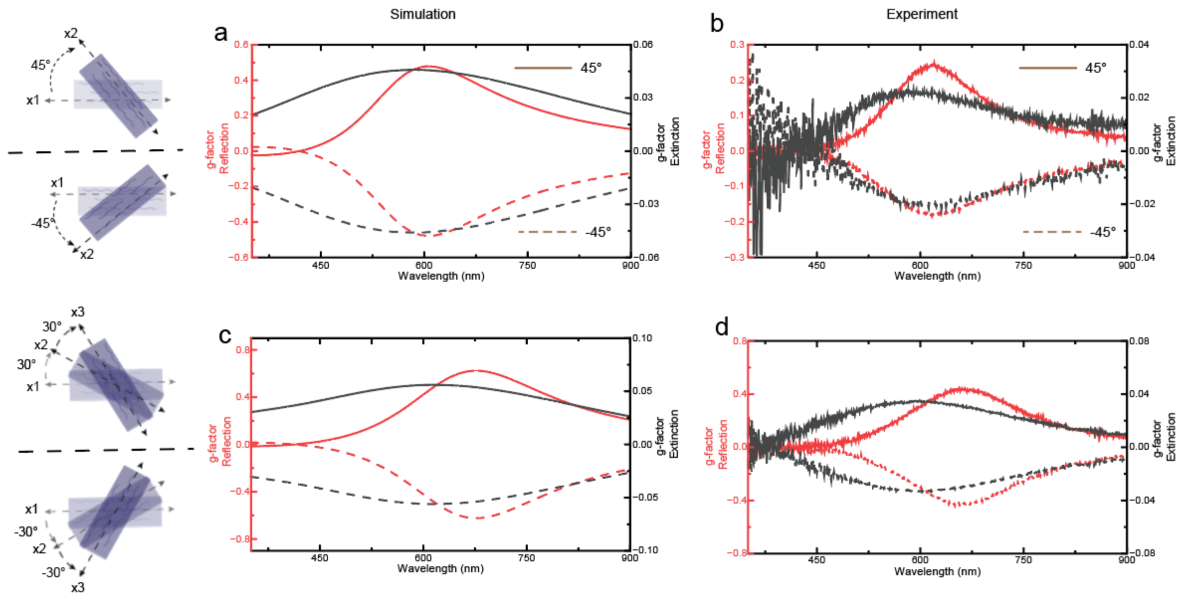

Figure S12. G-factor of twist films with different twist geometries. a, b Calculated g-factor from simulated (a) and experimental (b) chiral spectra of two-layer twisty stacked films. The thickness of a single film is 80 nm and twists at an angle of 45° or -45°. **c, d** Calculated g-factor from simulated (c) and experimental (d) chiral spectra of three-layer twisty stacked films. The thickness of a single film is 80 nm and twists at an angle of 30° or -30°. The scheme on the left of each panel shows the corresponding twist geometry.

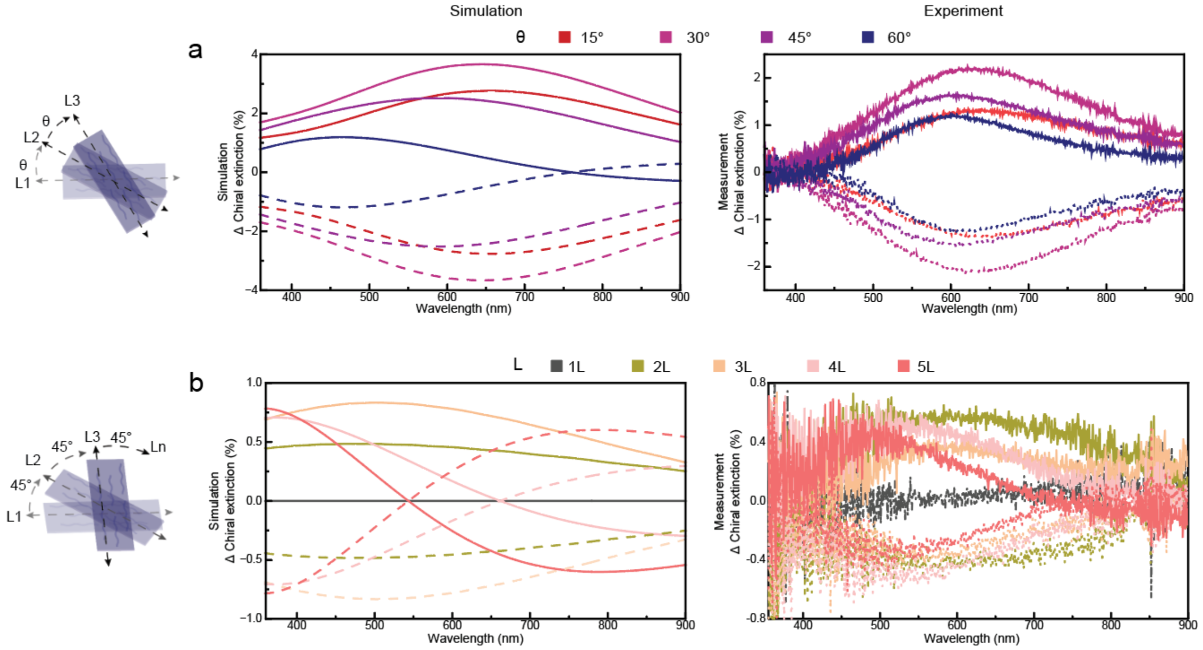

Figure S13: Tunable chirality. a FDTD simulated (left) and experimental measured (right) ΔExtinction intensity between RCP and LCP for three-layered samples with different twist angles. The thickness of each layer is 80 nm. **b** FDTD simulated (left) and experimental measured (right) extinction intensity difference between RCP and LCP with increasing layer numbers while keeping 45° twist between adjacent layers. The thickness of each layer was 40 nm.

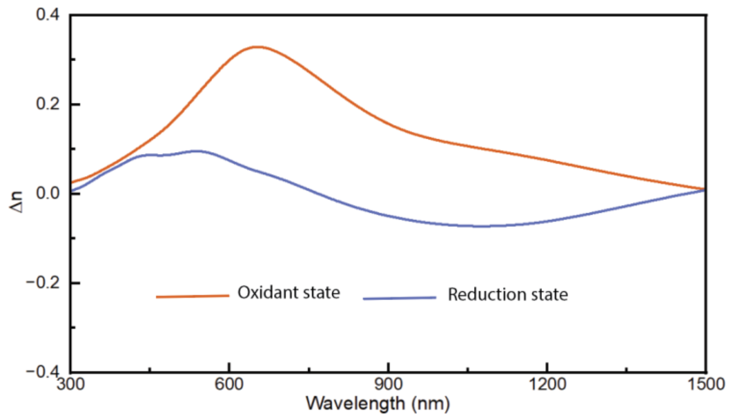

Figure S14: LB of a strained PEDOT:Sulf film before and after chemical reduction. The values were acquired by ellipsometry for a 25%-strained film (40 nm thickness) that was attached on a sapphire substrate.

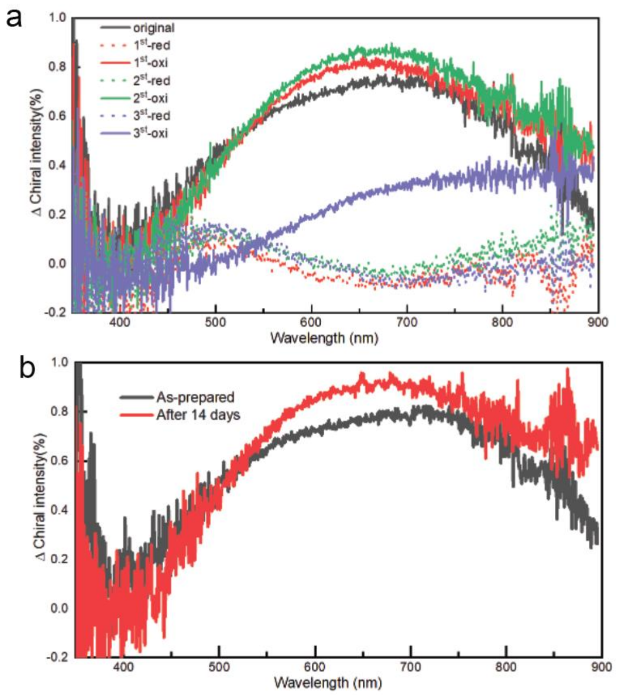

Figure S15. (a) Reversibility and (b) stability test for a two-layer twist-stacked system (45° and 80 nm layer thickness). The y-axis shows the chiral reflection intensity difference between RCP and LCP. For the reversibility test, the twist-stacked films were reduced by PEI vapor and re-oxidant by 1M sulfuric acid solution. The decrease of the CD values after three cycles is due to partial delamination of films from the substrate during the solution process. For the stability test, the twist-stacked films were measured two weeks after the preparation.

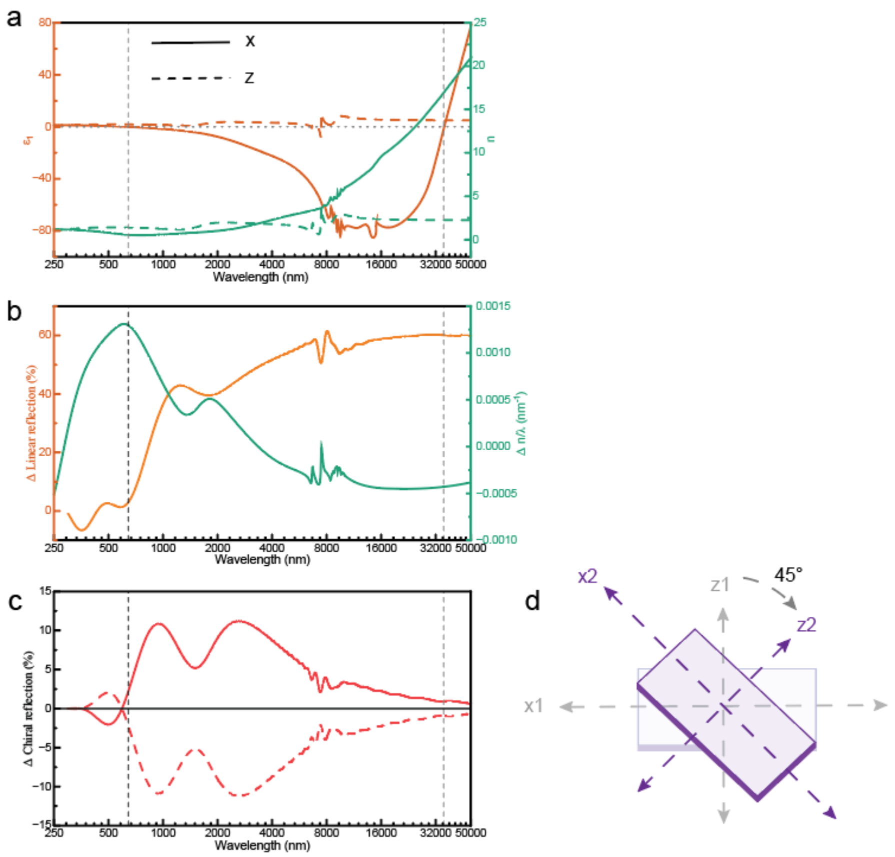

Figure S16: Simulated CD utilizing the out-of-plane hyperbolic property of PEDOT:Sulf films. a Real part of the in-plane (*x*) and out-of-plane (*z*) permittivity, and real refraction index, of as-grown PEDOT film by the VPP method. **b** Out-of-plane specific linear birefringence (Δn/λ) and linear reflection dichroism. The out-of-plane linear birefringence was calculated as $\Delta n = n_z - n_x$. **c** CD of two out-of-plane films twist-stacked at 45° and -45°, as depicted in **d**. The thickness of each film was 100nm. The vertical dashed lines indicate the plasma wavelength positions. The simulations show that the effective CD wavelength can be as large as from 0.4 to 50 μm because of the broad hyperbolic region of the material. Therefore, it is reasonable to predict that twist-stacking of well/aligned conductive polymers can generate effective CD with wavelength expanding from the visible to the far-infrared region.

Table S1: Comparison of chiroptical systems based on conjugated polymers.

Structure	Method	Thickness	CD bandwidth	*g*-factor
Aggregates of π-conjugated polymers	Polymer synthesis from chiral monomers[1]	Solution	Relates to the bandwidth of neutral state absorption bands (<300 nm)	~0.018 (absorption)
Fluorene Copolymers	Forming cholesteric liquid crystalline[2]	no value	Depends on the absorption or emission band (~100 nm)	0.001-0.8 (absorption, emission)
Achiral conjugated polymer-chiral small molecule additive blends	Self-assembly by using chiral small molecule as template[3]	~200 nm	Relates to the absorption band (<300 nm)	0.25–1.5 (absorption)
Hyperbolic highly conductive PEDOT thin films (this study)	Twist-stacking aligned conducting polymer thin films	~100 nm	Depending on the hyperbolic bandwidth. (Measured in this work: >500 nm; Predicted for improved alignment: several micrometers)	Measured: ≈0.5 (reflection in three layers) Predicted (for improved alignment): >1

References

[1] C. R. Grenier, S. J. George, T. J. Joncheray, E. Meijer, J. R. Reynolds, *Journal of the American Chemical Society* **2007**, 129, 10694.
[2] C. Kulkarni, M. H. C. van Son, D. Di Nuzzo, S. C. J. Meskers, A. R. A. Palmans, E. W. Meijer, *Chemistry of Materials* **2019**, 31, 6633.
[3] J. Wade, J. N. Hilfiker, J. R. Brandt, L. Liirò-Peluso, L. Wan, X. Shi, F. Salerno, S. T. Ryan, S. Schöche, O. Arteaga, *Nature communications* **2020**, 11, 6137.
